# Functional Autoreactive Anti-β2 Adrenergic Antibodies May Contribute to Insulin Resistance Profile in Patients with Chronic Chagas Disease

**DOI:** 10.3390/pathogens10030378

**Published:** 2021-03-21

**Authors:** Luz María Rodeles, Miguel Hernán Vicco, Álvaro Siano, Leonardo Andrés Fuchs, Luz María Peverengo, Silvia Sanchez Puch, Cora Beatriz Cymeryng, Iván Sergio Marcipar, Pablo Arias

**Affiliations:** 1 Laboratorio de Investigación en Salud, Centro de Estudios en Salud Global, Facultad de Ciencias Médicas, Universidad Nacional del Litoral, C3000 Santa Fe, Argentina; mvicco@fcm.unl.edu.ar (M.H.V.); fuchsleonardoandres@gmail.com (L.A.F.); imarcipr@gmail.com (I.S.M.); 2 Laboratorio de Péptidos Bioactivos, Departamento de Química Orgánica, Facultad de Bioquímica y Ciencias Biológicas, Universidad Nacional del Litoral, C3000 Santa Fe, Argentina; alvarosiano@gmail.com; 3 Laboratorio de Tecnología Inmunológica, Facultad de Bioquímica y Ciencias Biológicas, Universidad Nacional del Litoral, C3000 Santa Fe, Argentina; luzpeverengo@gmail.com; 4 Instituto de Investigaciones en Microbiología y Parasitología Médica, Universidad de Buenos Aires—Consejo Nacional de Investigaciones Científicas y Técnicas (CONICET), C1121 Buenos Aires, Argentina; ssp292@hotmail.com; 5 Laboratorio de Endocrinología Molecular (CEFYBO-CONICET), Facultad de Medicina, Universidad de Buenos Aires, C1121 Buenos Aires, Argentina; coracymeryng@gmail.com; 6 Cátedra de Fisiología Humana, Facultad de Ciencias Médicas, Universidad Nacional de Rosario, C2002 Rosario, Argentina; drpabloarias@hotmail.com

**Keywords:** adrenergic beta-2 receptor agonist, antibodies, chagas disease, insulin resistance, pituitary adrenocorticotropin-secreting cells, cyclic AMP

## Abstract

Potential activation of β2 adrenergic receptors (β2AR) by specific autoreactive antibodies (Abs) that arise during the host reaction to *Trypanosoma cruzi*, could contribute to the elevated prevalence of metabolic disturbances described in patients with chronic Chagas disease (CCD). This study aimed to determine the prevalence of anti-β2AR Abs in patients with CCD, as well as the correlation of these Abs with the presence of glucose and lipid metabolism disturbances, in order to explore their association with an insulin resistance profile. Additionally, we tested the functional effects of anti-β2AR Abs employing an in vitro bioassay with neuroendocrine cells expressing β2AR. A clinical and metabolic evaluation including an OGTT was performed in 80 CCD patients and 40 controls. Anti-β2AR Abs were measured by an in-house-developed ELISA, and the β2 adrenergic activity of affinity-purified IgG fractions from patient’ sera were assayed in CRE-Luc and POMCLuc transfected AtT-20 cells. A higher proportion of dysglycemia (72.5% vs. 37.5%; *p* = 0.001) was observed in the CCD group, accompanied by increased HOMA2-IR (*p* = 0.019), especially in subjects with Abs (+). Anti-β2AR Abs reactivity (7.01 (2.39–20.5); *p* = 0.0004) and age >50 years (3.83 (1.30–11.25); *p* = 0.014) resulted as relevant for IR prediction (AUC: 0.786). Concordantly, Abs (+) CCD patients showed elevated metabolic risk scores and an increased prevalence of atherogenic dyslipidemia (*p* = 0.040), as compared to Abs (−) patients and controls. On functional bioassays, Abs exerted specific and dose-dependent β2-agonist effects. Our findings suggest that anti-β2AR Abs may induce the activation of β2AR in other tissues besides the heart; furthermore, we show that in patients with CCD these Abs are associated with an insulin resistance profile and atherogenic dyslipidemia, providing biological plausibility to the hypothesis that adrenergic activation by anti-β2AR Abs could contribute to the pathogenesis of metabolic disturbances described in CCD patients, increasing their cardiovascular risk.

## 1. Introduction

Chagas disease is a parasitic infection caused by the protozoan *Trypanosoma cruzi* (*T. cruzi*) affecting about 8 million people mostly in Latin America. Nevertheless, during the last decades, its distribution has extended towards non-endemic areas such as United States, Canada, Europe, and even some Western Pacific countries due to migration flows. Considered a neglected tropical disease, it remains, however, a relevant public health problem causing more than 12,000 deaths per year and a high disability burden in affected areas [1].

Nearly 30% of chronically infected people develop cardiomyopathy, digestive disturbances (as megacolon or megaesophagus), or both. The heterogeneous clinical expression of the disease has been related to the broad spectrum of host–parasite interactions taking place during the chronic infection [2]. From this perspective, several mechanisms underlying chronic Chagas disease (CCD) pathogenesis have been described, including tissue damage and inflammatory response due to parasite persistence, autoimmunity, microvascular lesion, and autonomic dysfunction [3]. Despite disagreement arising in the past as to a possible preponderance of any of these mechanisms, nowadays, an integrative paradigm is accepted. Parasitic persistence is required for disease development, capable of gathering and maintaining a chronic inflammatory status that would intend to be controlled through mounting a particularly intricate immune response [4]. In this context, harmful mechanisms which are not restricted only to direct agent damage, but are also related to the degree of immune balance achieved, have become relevant [3].

Regarding the involvement of humoral autoimmune response, several autoreactive antibodies directed to the host’s mimetic antigens have been identified [5]. Among them, specific IgG antibodies cross-reacting with human β1 and β2-adrenergic receptors have been explored both in humans and in experimental models in relation to the pathogenesis of CCD cardiomyopathy [6]. Molecular mimicry with parasitic P0 protein has been proposed as the main origin of anti-β1 adrenergic antibodies that exert agonist effects on this type of receptor, inducing myocyte apoptosis [7]. Concerning the anti-β2 adrenergic receptor antibodies (anti-β2AR Abs), a similar mechanism has been described given the sequence homology between ribosomal proteins of *T. cruzi* and the H26Q peptide of the second extracellular loop of the human β2AR [8]. Moreover, parasite persistence triggers a chronic inflammatory subset, damaging host tissues and thus inducing the exposition of specific cryptic sequences that may turn into novel antigenic epitopes, a mechanism that could also induce the development of anti-β2AR Abs [9,10].

β2ARs are a receptor subtype naturally activated by catecholamines or other related molecules. It is encoded by the ADRB2 gene, located on chromosome 5 of the human karyotype, very close to the coding site for the α1 adrenergic receptor. Different polymorphisms, point mutations, or alterations in the regulation of this gene have been linked to the development of obesity, type 2 diabetes mellitus (DMT 2), and bronchial asthma (Hawkins et al., 2008) [11]. Physiologically, β2ARs play a fundamental role in providing the necessary resources (energy and blood supply) to execute the stress response orchestrated by the sympathetic nervous system and the hypothalamic–pituitary–adrenal (HPA) axis. For this reason, they present a wide tissue distribution. Some notable effects that occur after its activation include positive ino- and chronotropism, bronchodilation, mydriasis, decreased intestinal motility, thick salivary secretion, and smooth muscle relaxation. Regarding metabolic effects, their activation induces an increase in glycogenolysis and hepatic neoglycogenesis and greater lipolysis. In addition, it favors the pancreatic secretion of insulin [12].

We hypothesize that if these specific Abs are capable of activating the β2 adrenergic receptor in metabolically active tissues (i.e., liver, fat), people infected by *T. cruzi* that bear positivity for anti β2AR-Abs might express a metabolic profile different to those with negative results. 

A notably increased prevalence of obesity and diabetes was described in subjects with CCD [13,14]. In addition, in a retrospective cohort based on nutritional assessment, increased waist circumference, fasting hyperglycemia, and atherogenic dyslipidemia were found [15].

Inflammatory and metabolic changes induced by *T. cruzi* persistence in adipose tissue have been proposed as the main causative mechanisms of these metabolic disturbances. Increased systemic levels of pro-inflammatory cytokines (i.e., IL-6, MCP-1, TNF-α) were described in murine experimental models of infection in agreement with results obtained in infected adipocytes of 3TC-L1 cell line culture [16]. Besides, in vitro evidence of inhibition of PPAR-γ pathway, associated with diminished adiponectin synthesis and higher resistin levels, also contributed to clarifying these aspects [17]. Thus, complex immunoendocrine interactions take place during the chronic inflammatory response elicited by the parasite.

We hypothesize that if β2AR-Abs are capable of activating the β2 adrenergic receptor in metabolically active tissues (i.e., liver, fat), people infected by *T. cruzi* that bear positivity for anti β2AR-Abs might express a metabolic profile different to those with negative results. 

Therefore, this study aimed to determine the prevalence of anti-β2AR Abs by enzyme immunoassay in a sample of patients with CCD, as well as the correlation of these Abs with the presence of glucose and lipid metabolism disturbances in patients in order to explore their association with an insulin resistance profile. Additionally, a functional bioassay on a cell line model expressing native β2 adrenergic receptors employing affinity-purified IgG fractions from patients’ sera was performed in order to verify the effects of anti-β2AR Abs on another cell type different to cardiomyocytes.

## 2. Results

### 2.1. CCD Association with Increased Prevalence of Dysglycemia and Insulin Resistance

General features of the 120 included patients are summarized in Table 1. As compared to control subjects (CON), no significant differences were observed between the groups regarding age, sex, and BMI. 

As can be observed from Table 2, in the CCD group, a greater proportion of patients with IFG and/or IGT was found and the combination of these two dysglycemic states was more frequent, as well. Concordantly, individuals in this group showed moderate to high risk of type II diabetes development (Finnish Diabetes Risk Score (FINDRISC) >12 points) in 47.4% of cases, twice as high as in the CON group. Similarly, metabolic syndrome was observed in about 66% of patients with CCD, compared to 35% (*p* = 0.002) in the CON group. 

CCD subjects presented increased levels of fasting glucose (91.0 (84.0–99.5) vs. 105 (94.0–114); *p* < 0.001) and insulin (9.56 (7.18–14.6) vs. 15.15 (8.21–24.9); *p* = 0.009). In consequence, elevated HOMA2-IR values (reflecting hepatic IR) were found among CCD subjects. Besides, lower Matsuda index values (insulin sensitivity parameter) were observed in this group. Although there were no differences in the HOMA2-β% insulin-secretion indicator, an increased insulinogenic index was found between CCD patients; no statistically significant differences were found in the peripheral disposition (PIDI) of said hormone.

It should be mentioned that the CCD group has a slightly greater proportion of women with at-risk waist circumference, an issue that is considered in particular in the multivariate analyses.

In relation to the cardiovascular assessment, about ~40% of patients of both groups presented at least one risk factor. The percentage of hypertensive individuals in each group was similar, but dyslipidemia was more frequent in CCD patients (56.3% vs. 31.7%; *p* = 0.017). This was mainly due to higher levels of non-HDL cholesterol (140 ± 30.4 vs. 158 ± 50.5 mg/dL; *p* = 0.026). The atherogenic index of plasma was similar in both groups (0.596 (0.345–0.740) vs. 0.492 (0.429–0.601)).

### 2.2. Anti-β2AR Antibodies Reactivity in Patient Sera and Functional Bioassay

**Figure 1:**
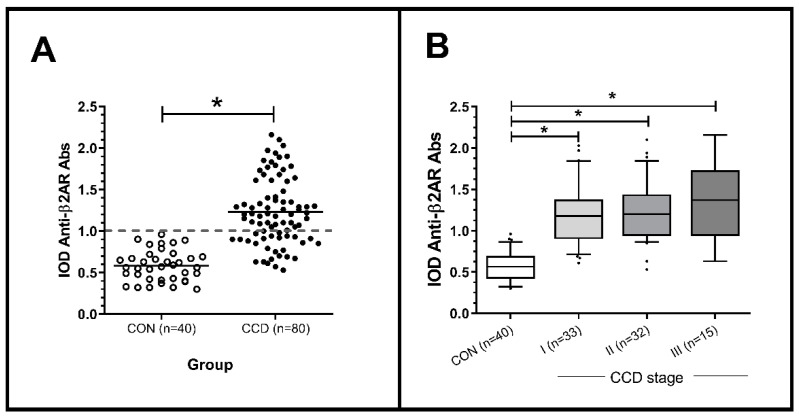
Anti-β2AR antibodies levels (**A**) in each group of patients through CCD stages (**B**). CCD: chronic Chagas disease; IOD: index of optic density. Grey dashed line indicates the corresponding value of IOD = 1 (cut off). (**A**) * Mann–Whitney U, *p* = 0.0001. (**B**) * Kruskal–Wallis test, *p* < 0.001.

Out of 80 CCD individuals, 70% (n = 56) showed positive results for anti-β2AR Abs with a mean IOD value of 1.43 (±0.318), as can be observed in Figure 1A. All CON subjects (n = 40) resulted negative for anti-β2AR Abs. Although higher Abs values were observed in patients in stage III of the Storino classification compared to those in stages I and II (1.35 (±0.487) vs. 1.27 (±0.393) and 1.23 (±0.370), respectively), this difference was not statistically significant (Figure 1B).

**Figure 2:**
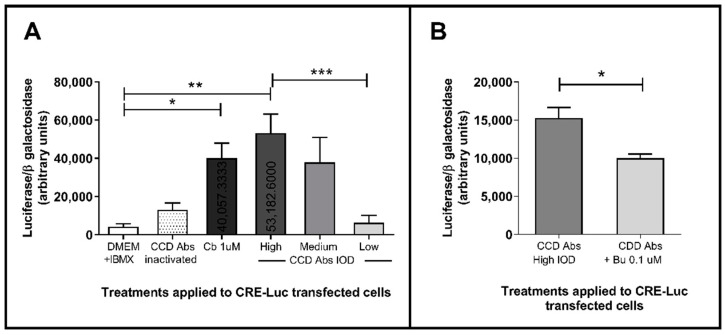
Effects of anti-β2AR antibodies of CCD patients on luciferase production in CRE-Luc transfected cells compared with β2AR agonist clenbuterol (**A**) and inhibition with selective antagonist butoxamine (**B**). DMEM+IBMX: control group only with basal medium and 3-isobutyl-1-methylxanthine (IBMX) as phosphodiesterase inhibitor; CCD Abs: antibodies of patients with chronic Chagas disease; IOD: index of optic density; Cb: clenbuterol; Bu: butoxamine. (**A**) Kruskal–Wallis test, *p* = 0.028; *, **, *** Mann–Whitney U, *p* = 0.010; 0.035; 0.013. (**B**) * Mann–Whitney U, *p* = 0.027.

**Figure 3:**
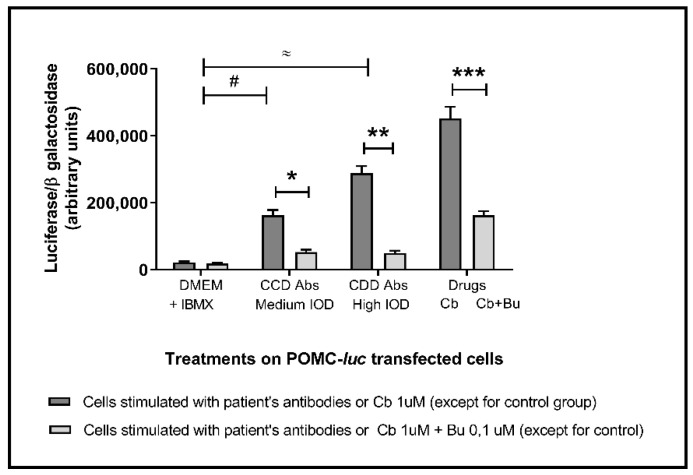
Specific inhibition of the agonistic effect exerted by anti-β2AR Abs in POMC-Luc transfected cells. Effect of Abs on luciferase expression compared to the result after incubation with the β2AR agonist clenbuterol and inhibition with the selective antagonist butoxamine. DMEM+IBMX: Control with basal medium only; CCD Abs: antibodies of patients with chronic Chagas disease; IOD: index of optic density; Cb: clenbuterol; Bu: butoxamine. *,**,*** Mann-Whitney U *p* = 0.0008, 0.0001 and 0.0002; ≈, # Mann-Whitney U *p* = 0.0004 and *p* = 0.001.

Regarding the functional bioassays, Figure 2 and Figure 3 present results obtained from the assessment of the effect of anti-β2AR Abs purified from patients on the activation of β2AR expressed on the surface of AtT-20 cells and their downstream cAMP-dependent signaling pathway. Studied antibodies were capable of inducing significant increases in normalized luciferase activity in relation to negative controls, both in CRE-Luc (*p* = 0.035) and POMC-Luc (*p* = 0.0002) transfected cells. These effects were similar to those elicited by stimulation with the β2-specific agonist Cb. The observed stimulation also showed dose-dependent characteristics through the three groups of purified anti-β2AR Abs (lower, middle, and upper IOD tertile). Otherwise, stimulation with heat-inactivated anti-β2AR Abs did not exert relevant stimulating effects, presenting similar levels to cells incubated only with the basal medium.

Furthermore, when incubated with Bu (specific β2AR antagonist) prior to treatment with anti-β2AR Abs, cells responded inversely, showing a significant decrease in normalized luciferase activity in each transfection condition (*p* = 0.027 and 0.0001, for CRE-Luc and POMC-Luc respectively). As can be observed, the antagonist effect of the Bu was similar between cells treated with Cb and anti-β2AR-Abs-exposed ones but it was more evident in the resulting product of the pathway as showed in POMC-Luc-transfected cells treated with anti-β2AR Abs than in the group transfected with CRE-Luc (Figure 3).

### 2.3. Anti-B2AR Antibodies in CCD Patients: Correlation with an Insulin Resistance Profile

**Figure 4:**
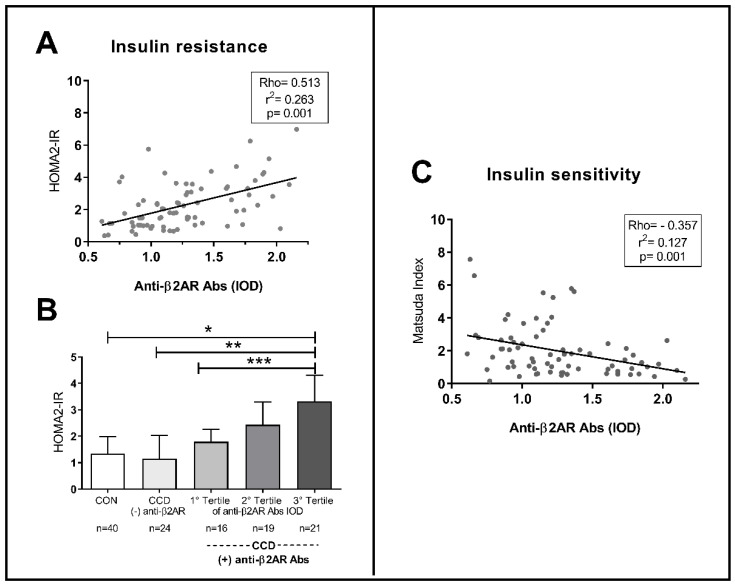
Relationship between the levels of Anti-β2AR antibodies with metabolic parameters of insulin resistance ((**A**,**B**), by HOMA2-IR) and sensitivity ((**C**), by Matsuda Index) in patients with chronic Chagas disease. The height of the bars indicates the median of the HOMA2-IR index for each group with the dispersion represented by the central vertical line (interquartile range). (**B**) Kruskal–Wallis test, *p* = 0.0001; *, **, ***: Mann–Whitney U, *p* = 0.0002; 0.020; 0.004.

Patients with (+) Anti-β2AR Abs showed higher HOMA2-IR values as compared to (−) anti-β2AR Abs ones. As can be taken from Figure 4A,B, anti-β2AR Abs levels present a positive correlation with HOMA2-IR (Rho: 0.50; *p* = 0.001). As seen in distribution by tertiles, IR was higher in those with clearly elevated Abs levels. On the other hand, as expected, anti-β2AR Abs evidenced an inverse correlation with Matsuda index, an insulin sensitivity parameter (Rho: −0.357; *p* = 0.001; Figure 4C). 

**Figure 5:**
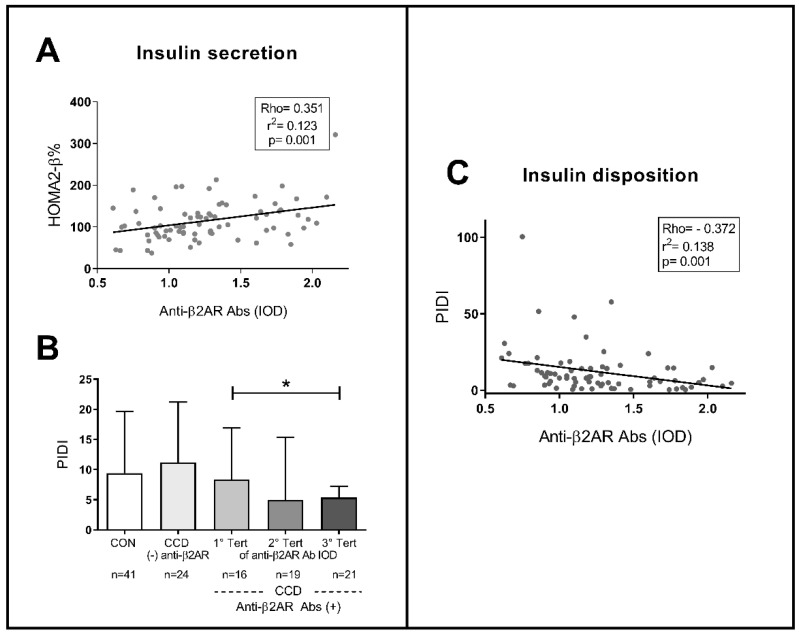
Relationship between levels of anti-β2AR antibodies and metabolic parameters of insulin secretion (**A**), by HOMA2-β) and peripheral disposition (**B**,**C**), by PIDI, in patients with chronic Chagas disease. The height of the bars indicates the median of the HOMA2-IR index for each group with the dispersion represented by the central vertical line (interquartile range). PIDI: peripheral insulin disposition index. (**B**) Kruskal–Wallis, *p* = 0.033; * Mann–Whitney U, *p* = 0.045.

Regarding insulin secretion, a mild positive correlation was found between anti-β2AR Abs and HOMA-β% (Rho: 0.351; *p* = 0.001; Figure 5A). As shown in Figure 5B,C, PIDI showed a significant negative correlation (Rho: −0.372; *p* = 0.001) in accord to the fact that patients with (+) anti-β2AR Abs showed lower PIDI values than (−) anti-β2AR Abs ones, with a progressive decrease through Abs tertiles.

There were no differences regarding age, sex, BMI, and the percentage of patients with at-risk waist circumference between subjects with (−) and (+) anti-β2AR Abs (Table 3). Nevertheless, higher proportions of individuals with FINDRISC score > 12 points, metabolic syndrome and combined dysglycemic states were found in the (+) anti-β2AR Abs group. These patients showed increased HOMA2-IR and HOMA2-β% values accompanied by diminished Matsuda index and PIDI. 

**Figure 6:**
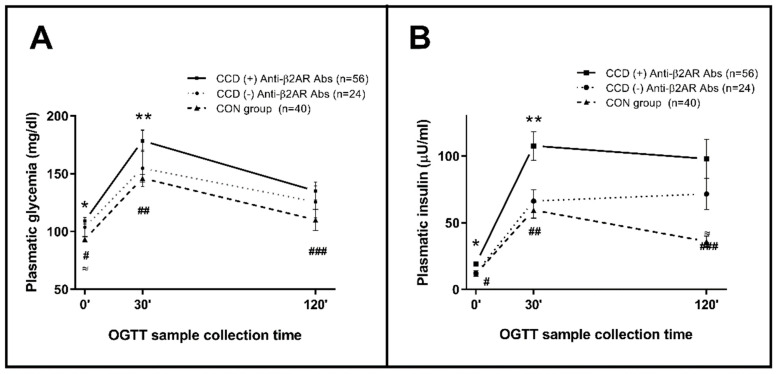
Glycemia (**A**) and insulinemia (**B**) levels throughout the Oral Glucose Tolerance Test performed in patients with Chagas disease (differentiated by serological reactivity to anti-β2AR) and controls. Measures of central tendency at each time are represented as median and interquartile interval. (**A**) Mann–Whitney U: *, ** Ac Anti-β2AR (+) vs. (−): *p* = 0.045; 0.044; ≈ Ac Anti-β2AR (−) vs. CON: *p* = 0.024; #, ##, ### Ac Anti-β2AR (+) vs. CON: *p* < 0.001; 0.001; 0.013. (**B**) Mann–Whitney U: *, ** Ac Anti-β2AR (+) vs. (−): *p* = 0.003; 0.015; ≈ Ac Anti-β2AR (−) vs. CON: *p* = 0.002; #, ##, ### Ac Anti-β2AR (+) vs. CON: *p* < 0.001; 0.002; 0.001.

Along the OGTT response, higher fasting glycemia and basal insulin levels, followed by augmented insulin secretion after 30 min were evidenced in CCD patients with (+) anti-β2AR Abs as compared to CCD individuals with (−) anti-β2AR Abs and to the CON group (Figure 6A,B). 

Regarding the lipid profile, a greater proportion of cases with dyslipidemia was detected among the (+) anti-β2AR Abs patients (51.7% vs. 12.5%; *p* = 0.004) with predominance of hypertriglyceridemia (58.9 vs. 33.3%; *p* = 0.035). The atherogenic index was also higher in this group (0.596 (0.345–0.740) vs. 0.492 (0.429–0.601); *p* = 0.040) compared to (−) anti-β2AR Abs group.

#### 2.3.1. Multivariate Exploration and Logistic Regression Analysis

**Figure 7:**
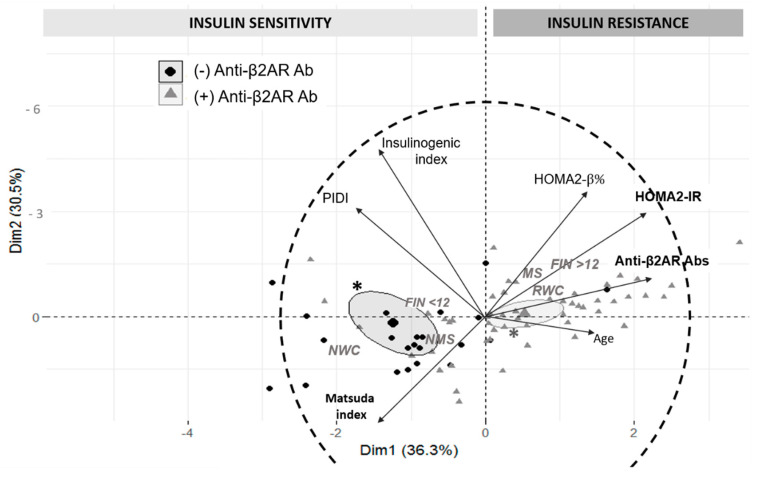
Variables factor map and individual principal component analysis in relation to studied metabolic parameters and anti-β2AR antibodies. The longitude of each vector segment indicates the representation of the variable on the principal component (more relevant when closer to the correlation circle—in dashed line). PIDI: peripheral insulin disposition index; RWC or NWC: at-Risk or normal waist circumference; MS or NMS: metabolic syndrome or negative for metabolic syndrome; FIN <12 or ≥12: FINDRISC Score <12 points or ≥12 points. *: confidence ellipses around clusters at 99% significance level.

In order to identify possible data patterns, principal components (PC) were obtained in relation to metabolic variables and anti-β2AR Abs levels (Figure 7). 

In concordance with previously described bivariate correlations, patients with higher anti-β2AR Abs levels show a notable trend to have increased IR, insulin secretion, and age as they are grouped in the upper-right quadrant following HOMA2-IR and HOMA2-β% vectors (PC 1). Conversely, those individuals with preserved insulin sensitivity are mostly distributed on the other side of the plot as can be observed in the vectors of PIDI and Matsuda index (PC 2), implying an inverse correlation with anti-β2AR Abs levels, particularly with the latter.

As can be taken from the confidence ellipses of each anti-β2AR Abs reactivity group (positive or negative), this condition would allow to significantly differentiate two main populations of individuals which present a particular distribution through the principal components (*p* = 0.010). Thus, patients with (+) anti-β2AR Abs (IOD ≥ 1) are located mostly in relation to PC 1 while negative patients would be principally situated along with PC 2. Although a certain degree of overlapping of patients with (+) anti-β2AR Abs can be observed in the insulin sensitivity quadrants, most of them present IOD values under the median in the CCD group (1.20 (0.941–1.57)).

Moreover, at-risk waist circumference (according to cut-off values by sex), FINDRISC score >12 points, and the presence of diagnostic criteria of metabolic syndrome were considered as qualitative supplementary variables. As expected, their distribution can be observed in the IR and higher anti-β2AR Abs quadrant.

#### 2.3.2. Logistic Regression Analysis

Subsequently, we applied a binary logistic regression model step-forward to predict IR considered as HOMA2-IR ≥ 1.90 (median value of CCD patients). Variables previously related to this outcome were included (i.e., age, IOD of anti-β2AR Abs, PWC by sex, FINDRISC score ≥12 points). To simplify the further application of the model, the continuous variables were categorized according to median values of their distribution in CCD patients. Therefore, 50 years was the selected cut-off for age while for the level of anti-β2AR Abs, the IOD value ≥1.20 was used. 

As stated above, other potential confounding variables were not included due to an insignificant bivariate association (i.e., smoking habit, balanced diet, and physical activity). 

As shown in Table 4, the analysis revealed that (+) anti-β2AR Abs and age ≥50 years contributed significantly to the prediction of IR (Hosmer and Lemeshow test of 3; *p* = 1). Internal validation by bootstrap sample (n = 80) yielded similar results. The logistic regression model composed of these two variables showed an AUC of 0.786 (95% CI: 0.676–0.873; *p* < 0.001) for IR prediction. Interaction of IR with other parameters as at-risk waist circumference categorized by sex or FINDRISC score ≥12 points, was not statistically significant to be retained by the model. 

The (+) LR of a patient with IR to present (+) anti-β2AR Abs was 2.51 for a patient with preserved insulin sensitivity. On the other hand, the (−) LR was 0.746, indicating a relevant remaining probability of presenting IR even with (−) anti-β2AR Abs.

## 3. Discussion

In the last decades, the clinical and research approach to *T. cruzi* infection has experimented with many changes that have led to a transition from the classical disciplinary perspective to a more integrative and systemic theory. This shift is supported by several investigations that have addressed different aspects of the intricate connections that are established between the immune and endocrine systems, employing a “common syntax” (chemical language) composed of different hormones, neurotransmitters, cytokines/chemokines, and the expression of functional receptors, which are shared by both systems [18].

In the case of *T. cruzi* infection, the situation is complex. On one hand, the hormonal status of the host modulates the immune response to the parasite; on the other, the latter can generate immunoendocrine disturbances that may have potentially harmful consequences for the host [19].

In this context, the present study assessed for the first time the potential association of autoreactive antibodies directed against β2AR, previously described in patients with CCD, with the presence of glucose metabolism disturbances. The first part of our study aimed to detect the presence of Abs in CCD patients employing an in-house enzyme immunoassay and to test their functionality on β2AR present in AtT-20 cells, a neuroendocrine bioassay system [20,21,22]. Although the immunopharmacological effects of these Abs as non-competitive allosteric agonists had already been tested in rat cardiomyocytes, they have not yet been evaluated in other cell lines [6]. Assays comparing the effect of anti-β2AR Abs from CCD patients with those elicited by specific β2-agonist and antagonist drugs were performed; they confirmed their binding capability to β2AR expressed in other tissues far beyond from the heart and the specific activation of the cAMP-dependent pathway.

Regarding the metabolic characterization of patients, this study describes an increased prevalence of metabolic syndrome in subjects with CCD as compared to the CON group (66.2% vs. 35.1%). Furthermore, higher fasting glycemia levels, augmented HOMA2-IR, and lower total insulin sensitivity (Matsuda Index) were detected, suggesting that both hepatic and peripheral components of IR might be more pronounced among CCD patients. This finding supports a previous observational study by Navarro et al. that prospectively included 74 individuals with asymptomatic CCD of whom 48% presented metabolic syndrome [15].

We observed a significant proportion of patients with dysglycemic states, doubling the frequency of the CON group for similar BMI, age, and sex distribution. These stages are defined by the presence of IFG and/or IGT implying a significative risk of progression to overt diabetes [23]. This is accentuated in those patients who present both conditions simultaneously, a situation that also occurs in a greater proportion among patients with CCD. However, its pathophysiological relevance is not limited only to the potential risk of diabetes, but to the fact that by themselves dysglycemic states constitute an independent cardiovascular risk factor as they are associated with the development of macrovascular events, especially at the coronary level, as well as higher mortality [24,25]. 

In addition, the FINDRISC score punctuation of sampled individuals indicates that 47% of the CCD group would be at moderate–high risk of developing diabetes. There are no previous reports of assessment of FINDRISC score in patients with CCD. Nevertheless, this instrument has been broadly validated and incorporated as a practical predictor of type 2 diabetes risk in the general population [26]. 

In relation to insulin secretion, despite basal levels (HOMA2-β%) being similar in both groups, CCD patients showed an increased insulinogenic index. It is important to consider the relevance of the latter as it is a dynamic parameter that comprises the insulin levels obtained after 30 min of the glucose load. This increment in the first phase of insulin secretion could be related to a chronic or persistent basal hyperglycemic state due to an increase in peripheral IR that might generate hyperstimulation of insulin secretion as a compensating mechanism to maintain glucose tolerance [27]. It has been proposed that this chronically increased glucose-stimulated insulin response is likely the most critical functional defect that influences the transition from pre-diabetes state to diabetes, as it contributes to progressive functional exhaustion of pancreatic β cells in their effort to maintain an adequate response to the hyperglycemia [28,29,30,31].

To this point, the results of the present study are consistent with the increased prevalence of obesity, metabolic syndrome, hyperglycemia, and diabetes in patients with CCD that has been suggested previously by other groups [13,14,15], adding new valuable information obtained from a more completely characterized sample in relation to clinical and metabolic parameters.

As the main underlying mechanism for the insulin resistance profile, it has been proposed that *T. cruzi* persistence in adipose tissue induces the adoption of a pro-inflammatory phenotype responsible for the metabolic disturbances that include increased production of pro-inflammatory cytokines (i.e., IL-6, MCP-1, TNF-α), inhibition of PPAR-γ pathway and diminished adiponectin synthesis with higher resistin systemic levels [16,17,32,33].

In addition, Onofrio et al. described that persistence of *T. cruzi* in an obesogenic environment, can contribute to immune metabolic imbalance. These authors observed IR during the acute phase of *T. cruzi* infection on a non-extreme obesity model developed in C57BL/6 mice, associated with hyperglycemia and hypoinsulinemia in the chronic stage, suggesting a feasible progression to diabetes during infection. Additionally, the model evidenced that the parasite promotes the induction of oxidative stress in visceral adipose tissue [34]. According to other studies performed in patients, this situation might be linked to the progression of CCD but also to other cardiovascular risk conditions as diabetes and atherosclerosis [35]. Related to this, our group has described that subjects with CCD presented increased epicardial adipose tissue thickness related to IR, diminished adiponectin plasmatic levels, and atherogenic dyslipidemia [36]. 

Regarding the potential role of anti-β2AR Abs, they resulted positive in ~70% of patients. A positive correlation between their level and hepatic IR (HOMA2-IR) was found, together with an inverse relation with peripheric insulin sensitivity (Matsuda index). Additionally, higher proportions of individuals with dysglycemic states (specially IFG), FINDRISC score ≥12 points and metabolic syndrome were found in patients with positive Abs. Furthermore, they present increased fasting glycemia and insulinemia levels; nevertheless, according to PIDI, this group showed a decrease in insulin disposition in peripheral tissues. It could be particularly relevant to consider that the β-cell regulates its insulin response to stimuli in relation to peripheral insulin sensitivity. PIDI takes into account the hyperbolic relationship between insulin first-phase response and insulin sensitivity index [37]; as a product of these two factors, this index provides an estimation of β-cell compensation ability that, according to our results, seems to be diminished in CCD patients with (+) anti-β2AR Abs.

Whereas the level of anti-β2AR Abs seems to be more related to parameters linked to the hepatic component of IR, we hypothesize that the interaction of studied Abs with the receptor expressed by hepatocytes could stimulate the adrenergic downstream pathway of these cells increasing glycogenolysis and gluconeogenesis that takes to higher glucose production. Resulting hyperglycemia would favor the development of an IR state that might require a compensatory effort of insulin production by β cells in a subset of low peripheral disposition of this hormone. This could explain the elevated fasting blood glucose values with a concomitant increase in basal insulin production and along the tolerance curve. 

Complementarily, it has been observed that in isolated mammalian pancreatic islets, despite a predominance of α2A effect, β2AR activation potentiates insulin secretion [38]. The increased insulin production observed in patients with higher anti-β2AR Abs (regarding HOMA-β% and insulinogenic index) could be also related to this. On the other hand, β2AR agonists induce lipolysis with increased release of glycerol from adipose tissue and skeletal muscle, mediating activation of hormone-sensitive lipase [39,40]. Likewise, they stimulate the generation of lactate by the latter tissue [41]. Both glycerol and lactic acid constitute the main substrates for hepatic gluconeogenesis, so the increase in glucose production could be enhanced through these pathways [42].

Finally, in lipidic metabolism, patients with (+) anti-β2AR Abs presented atherogenic dyslipidemia in a greater proportion, with a predominance of hypertriglyceridemia. This alteration is most frequent in patients with IR and type 2 diabetes [43]. Liver overproduction of VLDL is the main underlying disorder [44]; it depends on the availability of substrates (triglycerides and apoB100) derived from processes at the hepatic level (i.e., re-esterification of free fatty acids, de novo lipogenesis, and the absorption of remaining particles). Insulin is one of the hormones that regulate these processes. In patients with RI or diabetes, a defect in the regulatory capacity of insulin on ApoB secretion has been demonstrated, leading to increased VLDL release [45].

The above referred mechanisms could represent future perspectives of assessments derived from this work. It would be important to assay Abs effect on the liver or adipose tissue. In the same way, the agonist effect demonstrated in the culture of pituitary murine cells suggest a possible interaction between the autoreactive Abs and the function of the HPA axis, which could constitute another element that may intervene in the complex immunoendocrine imbalance that occurs during CCD that deserves further study.

It has been suggested that in the context of acute *T. cruzi* infection, this interaction between immune and endocrine systems is aimed at channeling host resources to face the elevated energy costs demanded by the immune response assembly. If a specific anti-parasitic treatment does not mediate, the disease progresses to the chronic phase and these same mechanisms, in the subset of a prolonged and exacerbated pro-inflammatory response, may contribute to generating an immunoendocrine environment which is potentially adverse for the host [7,46].

Considered as a whole, analysis of the data obtained from baseline and dynamic studies performed in patients with CCD suggests a marked profile of IR characterized by higher glycemic levels and increased insulin production with a concomitant decrease of the peripheral availability of this hormone. As can be seen, this profile is accentuated in the group of patients with positive anti-β2AR Abs. 

The metabolic disturbances that were described in CCD individuals could contribute to an increased metabolic risk of progression to type 2 diabetes mellitus as well as a higher cardiovascular risk profile. This issue may be particularly relevant since ~40% of patients with CCD have different degrees of cardiac affection due to the damage induced directly by the parasite and indirectly by the host immune response to its persistence [3]. The impact of IR, metabolic syndrome and/or dysglycemic states in terms of acceleration in the decline of functional capacity could be greater in CCD patients, particularly in those who already bear electrical and/or structural alterations. 

Although these hypotheses have never been tested in prospective studies and a cause–effect link cannot be established with certainty from a cross-sectional study, an association between an event (IR) and some explanatory variables could be a valuable starting point. CCD constitutes a permanent challenge not only because of its physio-pathological complexity but also because of its anchorage to intricate social and economic conditions that contribute to its perpetuation as a relevant health problem in many developing countries. With this background, it deserves an interdisciplinary and preventive approach directed to reduce adverse conditions, such as the increased risk of cardiovascular events, that may further deteriorate the quality of life of the people affected. 

## 4. Materials and Methods

### 4.1. Study Population and Subject Evaluation

A cross-sectional study was performed in *T. cruzi*-seropositive patients from Santa Fe, Argentina. Subjects from both sexes, older than 18 years, with a body mass index (BMI) between 18 and 30 kg/m^2^, were prospectively included between 2015 and 2019 at the Internal Medicine Department of the J. B. Iturraspe Hospital. Most of them were born in rural endemic areas for Chagas disease in the north of the country and were currently residing in non-endemic urban or peri-urban areas. Matched healthy individuals were included as control subjects in a 2:1 ratio (patients/controls).

The following exclusion criteria were considered:

- age ≥70 years;
- previously known diabetes mellitus, high hypertriglyceridemia levels (≥300 mg/dL), or other endocrinopathies;
- current treatment with drugs with known effects on carbohydrates metabolism (i.e., corticosteroids, thiazides, atypical antipsychotics, normo/hypoglycemic agents) and/or β adrenergic agonists/antagonists;
- pregnancy or immediate puerperium;
- chronic ethylism or relatively recent (≤2 years) abandonment of the habit.
- acute *T. cruzi* infection or chronic Chagas disease with advanced cardiac impact (i.e., severe arrhythmias, severe heart failure, evidence of acute decompensation);
- prior or present treatment with anti-*T. cruzi* compounds or immunosuppressive drugs;
- other relevant systemic complaints (i.e., autoimmune, oncological, or hematological diseases);
- psychiatric diseases or any other condition that might impair the patients’ capacity to give informed consent.

Besides complete clinical assessment, sampled individuals were subjected to 12-lead ECG recording, chest and abdominal X-rays, and Doppler echocardiography. Chronic Chagasic heart disease was characterized according to the three stages of damage proposed by Storino et al. (I: no evidence of heart injury; II: heart damage without heart failure; III: heart injury with systolic failure) [47]. 

After an overnight fast of at least 8 h, an Oral Glucose Tolerance Test (OGTT) was performed using a glucose load containing the equivalent of 75 g of anhydrous glucose dissolved in water [48]. Three samples were taken (at 0, 30, and 120 min) for glycemia and insulinemia measurements (sampling was always made between 8 and 10 a.m.) Blood samples were then treated with EDTA and centrifuged at 2000 rpm for 30 min to obtain plasma. Glycemic levels were determined using an enzymatic colorimetric method, and insulinemia was detected by electrochemiluminescence (Insulin^®^, Roche Diagnostics, Bassel, Switzerland) with a detection limit of 0.2 µU/mL. Fasting lipid levels (total cholesterol, HDL cholesterol, and triglycerides) were also determined by enzymatic colorimetric methods (Cholesterol Gen.2^®^, HDL-Cholesterol Plus 3rd Generation^®^, Triglycerides^®^, Roche Diagnostics, Bassel, Switzerland). LDL cholesterol levels were estimated using the Friedewald formula [49]. Samples were then stored at −80 °C for use in anti-β2AR Abs measurements and purification.

The HOMA2 model was employed to estimate IR (hepatic component) in basal conditions with the HOMA2-IR parameter, while HOMA2-%B was used for basal insulin secretion [50,51]. On the other hand, the means of the glycemic values during the OGTT are included in the Matsuda Index that represents mainly the peripheral muscle insulin sensibility (inverse of the total IR) [52]. Insulinogenic index was also assessed as a measurement of the first-phase (0–30 min) insulin response to glucose challenge, while peripheral insulin disposition index (PIDI) was employed to estimate insulin secretion in relation to peripheral IR degree [53]. 

According to the current guidelines, pre-diabetes state was assessed by the presence of impaired fasting glucose (IFG) and/or impaired glucose tolerance (IGT) [48]. In addition, the Finnish Diabetes Risk Score (FINDRISC) was calculated in order to assess the 10-year risk of developing type 2 diabetes [54]. The presence of dyslipidemia was defined by at least one of the following: total cholesterol levels ≥240 mg/dL, triglyceride levels ≥150 mg/dL, LDL cholesterol levels ≥140 mg/dL, HDL cholesterol levels <40 mg/dL (males) or <50 mg/dL (females), or the use of lipid-lowering drugs. Non-HDL cholesterol value (TC-HDL cholesterol) and the plasma atherogenic index (logarithm of the TG/HDL-c ratio) were estimated [55]. The latter is considered a risk predictor for atherosclerosis and coronary heart disease, reflecting the relationship between protective and pro-atherogenic lipoproteins.

The conduction of this study was carried out following the ethical principles for research with human beings established in the Nuremberg Code, the Belmont Report, and the Declaration of Helsinki of the World Medical Association. The protocol was registered and approved by the Provincial Bioethics Committee of the Province of Santa Fe (Protocol Register N°300) and by the Bioethics Committees of the UNL (Faculty of Medical Sciences and Faculty of Biochemistry and Biological Sciences). Patient care and all evaluations performed were conducted following the ICH Good Clinical Practice international guidelines and national requirements for clinical and epidemiological studies.

Every subject with the potential to participate in the study was informed about the characteristics of the study and potential risks; after that, a written copy of informed consent was given and signed by patients who voluntarily agreed to participate. The databases were designed and completed to ensure the coding of each patient to safeguard the confidentiality of their data, as well as the rest of the clinical and epidemiological information.

### 4.2. H26Q Peptide Synthesis and Assessment of Anti-β2AR Antibodies 

The H26Q peptide (H-W-Y-R-A-T-H-Q-E-A-I-N-C-Y-A-N-E-T-C-C-D-F-F-T-N-Q) was produced for use as a coating antigen for an in-house-developed indirect enzyme immunoassay. The sequence was previously reported and corresponds to 172 to 197 amino acid sequence of the second extracellular loop of the human β2AR [8,53]. It was synthesized as a C-terminal amide by Fmoc solid phase as per the same techniques described in a previous work [56].

Then, anti-β2AR Abs were measured in patient’s samples by an indirect immunoassay (ELISA) [57]. Briefly, microtiter plates were coated with 1:100 dilution of a 50 ug/mL H26Q peptide solution suspended in 1% β-mercaptoethanol and 0.05 M carbonate buffer, pH 9.6, and absorbed for 1 h at room temperature. After overnight incubation at 4–8 °C, plates were washed 5 times with 200 uL/well 1% phosphate-buffered saline solution (PBS) and 0.1% Tween^®^ 20. After that, 5% skim milk on 1% PBS was employed as blocking buffer during 1 h at 37 °C. Then, a wash cycle was performed and a 1:100 dilution of human serum in 1% skim milk on 1% PBS was placed on each well and incubated at 37 °C for 1 h. Peroxidase-conjugated goat antihuman Ig G (Chemicon, Merck KGaA, Darmstadt, Germany) was added after a new washing cycle (1:5000). Following the addition of 20 uL/well of trimethylbenzidine substrate, plates were read at 450 nm in an absorbance reader (BioTeK, Shoreline, WA, USA).

All serum samples were evaluated in duplicate, calculating the mean optical density value of these simultaneous assessments. In each plate, 6 samples from the control group (seronegative for *T. cruzi*) were also simultaneously assayed. For assay standardization, Abs levels were expressed as the Index of Optical Density (IOD) that results from the ratio between the optical density of the sample and the optical density of the negative standard cut-off (mean ± 2 SD). An IOD ≤1 was considered negative.

### 4.3. Specific Anti-β2AR Antibodies Purification 

A subset of positive samples for anti-β2AR Abs was randomly selected for purification. Specific anti-β2AR IgG Abs fractions were affinity purified in an activated Sepharose column (Cytiva, Marlborough, MA, USA) using H26Q peptide as coupling ligand. Elution fractions of ~500 uL were sequentially collected and tested again by ELISA for anti-β2AR Abs presence. Abs (+) fractions were divided into three groups according to the anti-β2AR Abs IOD (high, medium, and low IOD tercile) and conserved at −80 °C until they were used for cell stimulation in functional experiments. 

### 4.4. Functional Bioassay

#### 4.4.1. Cells and Culture Conditions 

For this purpose, a murine ACTH-secreting pituitary adenoma cell line named AtT-20/D16v-F2 (AtT-20) was obtained from the American Type Culture Collection [58]. Cells were maintained in DMEM (Invitrogen, Waltham, MA, USA) supplemented with 10% heat-inactivated fetal bovine serum, 100 U/mL penicillin, 100 g/mL streptomycin, and incubated at 37 °C and 5% CO_2_. 

To evaluate whether purified Abs exert a β2-AR agonist functional effect, we designed a comparative experiment using selective β2-AR drugs. In order to compare results with those obtained after stimulation with purified Abs, clenbuterol (Sigma-Aldrich, St. Louis, MO, USA) and butoxamine (Sigma-Aldrich, St. Louis, MO, USA) served as specific B2-AR agonist and antagonist, respectively. Stock solutions of both drugs were prepared by dissolving them into a final concentration of 10 mM in ethanol and PBS, respectively. 

For each experiment, purified Abs or drugs were diluted in a basal medium composed of serum-free DMEM with 200 uM/well 3-isobutyl-1-methylxanthine (IBMX) as phosphodiesterase inhibitor reaching a final volume of 100 uL/well. The optimum concentration of drugs was obtained in a dose-response assay with dilutions ranging from 0.1 to 10 μM. For clenbuterol (Cb), the selected dose was 1 uM whereas for butoxamine (Bu) it was 0.1 uM (Supplementary Figure S1A,B). 

In the case of purified Abs, dose response was tested using the described above IOD groups in a 1:3 dilution with a basal medium. Two conditions were employed as negative controls: (a) a heat-inactivated fraction of purified Abs from a patient of the CCD group, (b) only basal medium (DMEM+IBMX). Each treatment was applied in four replicates.

#### 4.4.2. Transfections and Reporter Assays

To assess the potential effects of purified antibodies on cAMP-dependent pathway, we transfected AtT-20 cells with DNA corresponding to the cAMP response element binding protein plasmid pCRE-Luc (0.10 g/well; kindly supplied by Eduardo Cánepa (FCEyN, UBA, CABA, Argentina); a luciferase reporter gene was employed. Additionally, POMC-Luc plasmid (0.18 g/well; provided by Domenico Accili (Addgene NCBI number 17553) was used to evaluate the consequent effects of the purified Abs on POMC production. For measurement of transfection efficiency, cells were co-transfected in both experiments with pCMV-βgal (0.02 g/well; Clontech (Mountain View, CA, USA). 

Cells were first seeded in 96-well plates (8 × 104 cells/well) supplemented with 10% bovine fetal serum and antibiotics (100 uL/well) for 24 h at 37 °C.

Then they were transfected with the above referred plasmids using Lipofectamine 2000 (Invitrogen, Waltham, MA, USA) following the manufacturer’s instruction.

Two days after transfection, cells were treated accordingly with drugs and/or antibodies from patients. Proportional luciferase activity obtained after 4 h of stimulation with purified Abs was compared with results after treatment with Cb for the same period. The antagonistic effect of Bu was evaluated in Abs- or Cb-treated cells after 30 min of preincubation with the former drug. Luciferase activity was determined with the Steady-Glo Luciferase Assay System (PromegaCorp, Madison, WI, USA) using a luminometer. Values were normalized to β-galactosidase activity measured with β-Galactosidase Assay Kit (Invitrogen, Waltham, MA, USA) microplate luminescence reader at 570–595 nm.

### 4.5. Statistical Analysis

Data were analyzed with MedCalc 12.2.1 (MedCalc Software Ltd., Ostend, Belgium) and GraphPad 5.03 (GraphPad Software, San Diego, CA, USA). The distribution of continuous variables was tested using the Kolmogorov–Smirnov test. Normally distributed continuous variables are expressed as means (±SD). Student’s non-paired t-test or one-way ANOVA (Student–Newman–Keuls test for all pairwise comparisons), as considered appropriate, were used to compare means. 

For variables not normally distributed, values were expressed as median (interquartile range). Non-parametric tests were employed for means comparisons (Mann–Whitney U, Kruskall–Wallis test). Pearson’s or Spearman’s correlation coefficient and adjusted R2 were determined for estimating the association between two quantitative variables. Chi-squared or Fisher’s exact tests were used for comparing categorical variables.

For multivariate data exploration, principal component analysis (PCA) of metabolic parameters and anti-β2AR Abs was conducted to assess their potential relevance and interrelation [59]. An eigenvalue >1 was considered as the cut-off point for determining the number of components finally included. 

Then, the association of main variables with IR parameters was studied. A multiple binary logistic regression model step forward was performed combining variables that were correlated to IR (in both bivariate and multivariate analyses). Positive and negative Likelihood Ratios (LR) were calculated. Then, we applied the bootstrap method to make an internal validation of the model in half of the sampled individuals. In addition, we plotted the discriminating capacity of the antibody levels to predict this endpoint in a receiver operating characteristic (ROC) curve. In all cases, a *p*-value <0.05 was considered significant.

## 5. Conclusions

A higher proportion of subjects with dysglycemic states (specially IFG), metabolic syndrome, and FINDRISC scores related to moderate and high risk of developing diabetes were observed in CCD group, especially in those with reactivity to anti-β2AR Abs. In agreement, an increase in the values of HOMA2-IR was observed, accompanied by a decrease in insulin sensitivity and peripheral disposition. Atherogenic dyslipoprotenemia was also more frequent in this group. The level of anti-β2AR Abs in CCD patients was related to an insulin-resistant profile, mainly involving the hepatic component of IR. On the other hand, the chronic inflammatory state produced by the persistence of *T. cruzi* would generate an immunoendocrine environment tending also to increase IR, especially at the peripheral level.

Regarding functional bioassays, anti-β2AR Abs presented the capability to exert a specific, dose-dependent agonistic effect on β2AR expressed in neuroendocrine cells.

Altogether, these results lend biological plausibility to the hypothesis that anti-β2AR Abs may have a concurrent role in the pathogenesis of metabolic abnormalities (i.e., hyperglycemia, insulin resistance, dyslipoprotenemia) described in patients with CCD, potentially increasing their cardiovascular risk.

## Figures and Tables

**Table 1:** General characteristics of sampled individuals.

	CON (n = 40)	CCD (n = 80)	*p*
Age (years) ^#^	44.6 ± 12.8	49.1 ± 10.1	ns
Sex (F/M)	21/19	47/33	ns
At-risk waist circumference (%)	95.5 ± 14.5	98.7 ± 12.1	ns
M (% ≥94 cm)	73.7% (n = 14)	81.3% (n = 26)	ns
F (% ≥80 cm)	66.6% (n = 14)	89.4% (n = 42)	0.037 *
BMI (kg/m^2^; RI) ^≈^	25.8 (23.9–28.3)	27.7(25.1–29.2)	ns
Underweight (%)	5.00	5.00	ns
Normal (%)	35.0	32.5	ns
Overweight (%)	60.0	62.5	ns

Results expressed as: ^#^ mean ±SD; ^≈^ median (IQR). ns: non-significant; * χ^2^. BMI: body mass index.

**Table 2:** General characteristics of sampled individuals.

	CON (n = 40)	CCD (n = 80)	*p*
FINDRISC Score (% ≥12 p)	20.0% (n = 8)	47.4% (n = 37)	0.039 *
**Dysglycemic states**			
IFG and/or ITG (%)	37.5 (22.5/20)	72.5 (62.5/31.3)	0.001 *
Combined IFG and ITG (%)	7.60 (n = 19)	20.8 (n = 26)	0.020 *
Metabolic Syndrome (%)	35.1% (n = 14)	66.2% (n = 53)	0.002 *
**Insulin resistance**			
HOMA2-IR ^≈^	1.28 (0.940–1.98)	1.90 (1.05–3.30)	0.019 ^‡^
Matsuda index ^≈^	2.82 (1.75–3.83)	1.49 (0.876–2.59)	<0.001 ^~^
**Insulin secretion**			
HOMA2-%β ^#^	113 ± 25.1	114 ± 47.7	ns
Insulinogenic index ^≈^	0.508 (0.292–1.11)	1.03 (0.476–1.74)	0.004 ^~^
Peripheral disposition (PIDI) ^≈^	5.13 (2.61–9.03)	7.95 (3.56–14.8)	ns

Results expressed as: ^≈^ mean ± SD; ^#^ median (IQR). The statistical test performed is indicated with a specific symbol if the result is statistically significant: * Fisher’s exact test; ^‡^ Student’s t; ^~^ Mann–Whitney *U*; ns: non-significant. IR: insulin resistance; PIDI: peripheral insulin disposition index.

**Table 3:** Clinical and metabolic features of patients with chronic Chagas disease, according to anti-β2AR antibody status.

	Anti-β2AR Antibody Status		*p*
	Negative (n = 24)	Positive (n = 56)	
Age (years) ^#^	46.6 ± 10.5	49.7 ± 9.46	ns
Sex (F/M)	15/9	31/25	ns
Waist circumference			
M (% ≥94 cm)	33.3% (n = 3)	39.2% (n = 10)	ns
F (% ≥82 cm)	60.0% (n = 9)	90.3% (n = 28)	ns
BMI (kg/m^2^; RI) ^≈^	26.8 (23.4–29.5)	28.1 (25.6–29.1)	ns
FINDRISC Score ^#^	8.30 ± 4.71	11.1 ± 4.97	0.028 ^‡^
FINDRISC Score (% ≥12 p)	31.8% (n = 6)	54.0% (n = 31)	0.025 *
Dysglycemic states (IFG or ITG)	70.8% (n = 17; 54.1%/29.1%)	78.5% (n = 44; 69.6%/39.2%)	ns
Combined IFG and ITG	8.30% (n = 2)	26.7% (n = 15)	0.042 *
Metabolic syndrome	45.8% (n = 11)	75.0% (n = 42)	0.014 *
Fasting glycemia (mg/dL) ^≈^	100 (91.5–107)	110 (94.7–115)	0.046 ^~^
Fasting insulin (µU/mL) ^≈^	8.84 (6.65–15.6)	15.5 (10.4–25.6)	0.006 ^~^
HOMA2-IR ^#^	1.61 ± 1.33	2.54 ± 1.41	0.010 ^‡^
Matsuda index ^≈^	2.14 (1.25–2.82)	1.22 (0.724–2.09)	0.030 ^~^
HOMA2-β% ^#^	95.1 ± 40.9	122 ± 48.1	0.021 ^‡^
Insulinogenic index ^≈^	0.869 (0.487–1.81)	1.20 (0.470–1.82)	ns
PIDI ^≈^	11.21 (5.42–21.2)	5.91 (2.88–14.2)	0.024 ^~^

Results expressed as: ^#^ mean (±DS); ≈ median (IQR). * χ^2^; ^‡^ Student’s t; ^~^ Mann–Whitney U; ns: non-significant. BMI: body mass index; IR: insulin resistance; IS: insulin sensitivity; ISI: insulin sensitivity index; PIDI: peripheral insulin disposition index.

**Table 4:** Significant variables for insulin resistance prediction in CCD patients from logistic regression model.

Retained Variables	OR (95% CI)	*p*
Age ≥ 50 years	3.83 (1.30–11.25)	0.014
(+) Anti-β2AR Abs (IDO ≥ 1.20)	7.01 (2.39–20.5)	0.0004
**Model AUC:** 0.786	(95% CI: 0.676–0.873)	*p* < 0.001

Discarded variables: At-risk waist circumference by sex (*p* = 0.575); FINDRISC score >12 points (*p* = 0.493).

## Data Availability

The data that support the findings of this study are available on request from the corresponding author. The data are not publicly available due to privacy or ethical restrictions.

## Supplementary Materials

The following are available online at https://www.mdpi.com/2076-0817/10/3/378/s1. Figure S1: Dose-response effect of clenbuterol and butoxamine on β2 adrenergic receptors ex-pressed by AtT20-cells.
