# Supplementary material for: Functional Autoreactive Anti-β2 Adrenergic Antibodies May Contribute to Insulin Resistance Profile in Patients with Chronic Chagas Disease
Source: Pathogens. 2021 Mar 21;10(3):378. doi: 10.3390/pathogens10030378 (PMC8004215; doi:10.3390/pathogens10030378)
Supplement: Supplementary file 1 [file pathogens-10-00378-s001.pdf]

Supplementary figure. Dose-response effect of clenbuterol and butoxamine on β2 adrenergic receptors expressed by AtT20-cells.

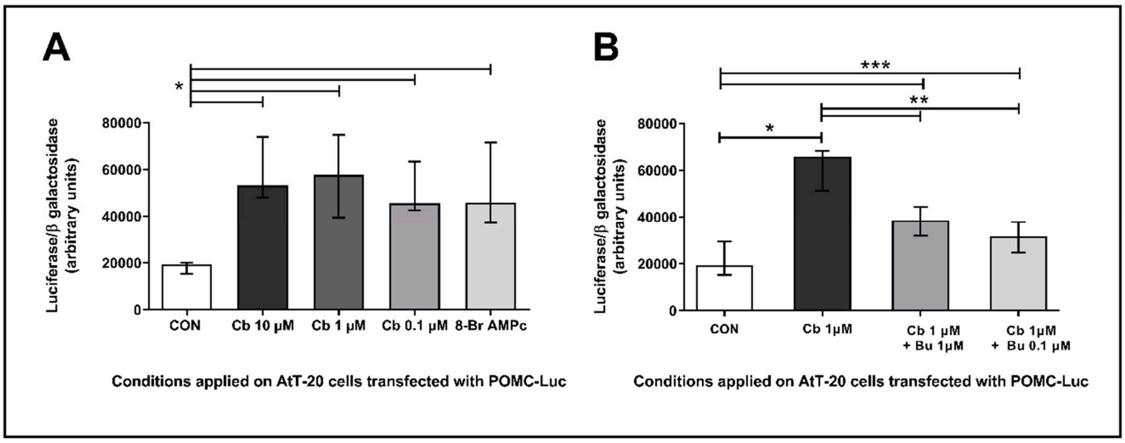

Results from experiments performed on AtT20 cells assessing the β2AR response to different concentrations of clenbuterol (Cb) and butoxamine (Bu). Drugs were prepared in basal medium (CON) composed of serum-free DMEM and IBMX in dilutions ranging from 0.1 to 10 μM. For Cb (A), selected dose was 1 μM whereas for Bu it was 0.1 μM (B). 8-Br cAMP is a cAMP permeable analog incorporated as positive control of cAMP-dependent signaling pathway.

(A)* Kruskal-Wallis: $p = 0.009$; (B) *** Kruskal-Wallis: $p < 0.001$ (CON vs. all conditions); Mann Whitney U: * $p = 0.028$ (CON vs. Cb 1 μM); ** $p = 0.018$ (Cb 1 μM vs. inhibitor treatment with Bu).
